# Hyperosmolar Hyperglycemic State As the Initial Presentation of Type 1 Diabetes Mellitus in an Adolescent: A Case Report

**DOI:** 10.7759/cureus.116797

**Published:** 2026-09-23

**Authors:** Ali T Alamer, Sajjad M AlKadhem, Osama Kattih, Ahmed A Al-Amoudi, Aida S AlJabri, Zainab M Almeftah, Husain A Althani

**Affiliations:** 1 Pediatric Intensive Care Unit, Almoosa Specialist Hospital, Al-Ahsa, SAU; 2 Pediatric Intensive Care Unit, King Fahad Medical City, Riyadh, SAU; 3 Pediatric Critical Care Medicine, Almoosa Specialist Hospital, Al-Ahsa, SAU; 4 Pediatric Endocrinology, Almoosa Specialist Hospital, Al-Ahsa, SAU; 5 Pediatrics, Almoosa Specialist Hospital, Al-Ahsa, SAU

**Keywords:** c-peptide, gad65 antibody, hyperosmolar hyperglycemic state, pediatric diabetes, severe hyperglycemia, type 1 diabetes mellitus

## Abstract

Hyperosmolar hyperglycemic state (HHS) is an uncommon but life-threatening hyperglycemic emergency in children and is more often associated with type 2 diabetes mellitus. We report a 13-year-old boy with a lean habitus and no previously diagnosed chronic illnesses who presented after three weeks of polyuria, polydipsia, fatigue, and weight loss. On admission, serum glucose was 1,080 mg/dL (60 mmol/L), hemoglobin A1c was 13.8%, measured serum osmolality was 331 mOsm/kg, calculated effective osmolality was 324 mOsm/kg, venous pH was 7.29, bicarbonate was 23.1 mmol/L, beta-hydroxybutyrate was 0.7 mmol/L, and urine ketones were negative. The biochemical profile met pediatric HHS criteria without significant ketoacidosis. He received an initial 20 mL/kg bolus of 0.9% sodium chloride followed by controlled deficit replacement. Intravenous insulin at 0.05 units/kg/hour was deliberately delayed until four hours after the initiation of fluid therapy, when the decline in glucose with fluids alone slowed to <50 mg/dL/hour. Glucose and effective osmolality then declined in a controlled fashion, while measured sodium rose gradually as hyperglycemia corrected; dextrose was added when glucose reached 275 mg/dL. No neurologic, thrombotic, renal, or major electrolyte complications occurred. Strongly positive GAD65 antibodies and a low C-peptide concentration obtained during concurrent hyperglycemia supported autoimmune type 1 diabetes mellitus (T1DM). The patient transitioned to subcutaneous basal-bolus insulin and was discharged on day 3. At three months, hemoglobin A1c was 7.0%. This case illustrates that HHS may be the first manifestation of autoimmune T1DM even in a lean adolescent and emphasizes the importance of interpreting glucose, sodium, and effective osmolality together while prioritizing controlled fluid replacement before insulin.

## Introduction

Type 1 diabetes mellitus (T1DM) is an immune-mediated disorder characterized by progressive pancreatic beta-cell destruction and loss of endogenous insulin secretion [1]. Diabetic ketoacidosis (DKA) is the most recognized acute metabolic emergency at diagnosis in children with T1DM. In contrast, hyperosmolar hyperglycemic state (HHS) is uncommon in pediatrics and is defined by extreme hyperglycemia and hyperosmolality with profound dehydration and minimal or absent ketoacidosis. Pediatric HHS is clinically important because fluid deficits are generally larger than those in DKA, and complications may include circulatory compromise, electrolyte disturbances, rhabdomyolysis, thrombosis, acute kidney injury, seizures, and cerebral injury [2-5].

Although HHS is classically associated with obesity and type 2 diabetes mellitus, it can occur at the onset of autoimmune T1DM. Because management differs from conventional DKA, particularly with respect to the magnitude of fluid replacement, delayed low-dose insulin, and close attention to sodium and tonicity, correct recognition of the phenotype is essential [2,4]. We report HHS as the initial presentation of autoimmune T1DM in a lean adolescent and highlight the diagnostic and therapeutic features that distinguish HHS from conventional DKA.

## Case presentation

A 13-year-old boy with no known chronic illness presented to the ED with a three-week history of progressive polyuria, polydipsia, nocturia, fatigue, and unintentional weight loss. He reported increased intake of sugar-sweetened beverages during the symptomatic period. There was a family history of T1DM and autoimmune hypothyroidism on the maternal side. He had no fever, vomiting, abdominal pain, altered consciousness, medication exposure, or previous hyperglycemic episodes.

On arrival, his weight was 35.3 kg and height was 158 cm (BMI 14.1 kg/m²), consistent with a lean habitus. Heart rate was 118 beats/min, blood pressure was 102/64 mmHg, respiratory rate was 20 breaths/min, temperature was 36.7°C, and oxygen saturation was 99% on room air. He was alert and fully oriented, with a Glasgow Coma Scale score of 15/15. Examination showed dry mucous membranes, sunken eyes, reduced skin turgor, cool distal extremities, and a capillary refill time of approximately 3 seconds, consistent with an estimated 10%-12% fluid deficit. There was no Kussmaul breathing, focal neurologic deficit, or clinical evidence of cerebral edema. Bedside capillary glucose exceeded the device measurement range.

Diagnostic assessment

Initial venous blood gas analysis showed a pH of 7.29, PCO₂ of 50 mmHg, and bicarbonate of 23.1 mmol/L. Serum glucose was 1,080 mg/dL (60 mmol/L), hemoglobin A1c was 13.8%, sodium was 132 mmol/L, potassium was 4.8 mmol/L, chloride was 94 mmol/L, phosphate was 1.22 mmol/L, magnesium was 0.82 mmol/L, blood urea nitrogen was 7.0 mmol/L, and creatinine was 88 µmol/L. Serum beta-hydroxybutyrate was 0.7 mmol/L, lactate was 2.2 mmol/L, and urine ketones were negative. Measured serum osmolality was 331 mOsm/kg.

Calculated effective serum osmolality was 324 mOsm/kg: 2 × measured Na (132 mmol/L) + glucose (60 mmol/L). Using a sodium correction factor of 1.6 mmol/L for each 100 mg/dL increase in glucose above 100 mg/dL, corrected sodium was approximately 148 mmol/L. The anion gap was approximately 15 mmol/L. Thus, the patient met the pediatric biochemical criteria for HHS: glucose >600 mg/dL, venous pH >7.25, bicarbonate >15 mmol/L, minimal ketonemia/ketonuria, and effective osmolality >320 mOsm/kg (Table 1) [2].

**Table 1:** Admission findings compared with pediatric HHS criteria. HHS: Hyperosmolar Hyperglycemic State.

Parameter	Patient result	Typical HHS criterion	Interpretation	Reference range
Random serum glucose	1,080 mg/dL (60 mmol/L)	>600 mg/dL	Meets criterion	≥200 mg/dL with classic symptoms supports diagnosis of diabetes
Venous pH	7.29	>7.25	Minimal acidemia	7.35-7.45
Bicarbonate	23.1 mmol/L	>15 mmol/L	No significant ketoacidosis	21-29 mmol/L
Beta-hydroxybutyrate	0.7 mmol/L	Minimal/absent ketonemia	Low	<0.5 mmol/L
Urine ketones	Negative	Small or absent	Meets criterion	Negative
Effective osmolality	324 mOsm/kg	>320 mOsm/kg	Meets criterion	275-295 mOsm/kg
Measured Na	132 mmol/L	Variable	Translocational hyponatremia	135-145 mmol/L
Corrected Na (1.6 factor)	≈148 mmol/L	-	Underlying hypertonicity	135-145 mmol/L

Management

The child was admitted to the pediatric intensive care unit for controlled correction of hyperosmolality and close neurologic and biochemical monitoring. Because perfusion was impaired but blood pressure was preserved, he received 0.9% sodium chloride at 20 mL/kg (706 mL) over the first hour. A fluid deficit of approximately 12% was estimated (about 4.2 L). After the initial bolus, 0.45% sodium chloride was used for controlled replacement of the remaining deficit plus maintenance requirements over approximately 48 hours, initially at about 150 mL/hour and subsequently adjusted according to hemodynamic status, urine output, measured sodium, glucose, and effective osmolality.

Fluids alone reduced serum glucose from 1,080 to 800 mg/dL over the first four hours. During the fourth hour, the glucose decline slowed to 35 mg/dL/hour; therefore, regular insulin was started at 0.05 units/kg/hour rather than at presentation. Once urine output was established, potassium was added at 40 mmol/L (20 mmol/L potassium chloride plus 20 mmol/L potassium phosphate) and subsequently adjusted according to serial potassium and phosphate concentrations. When serum glucose reached 275 mg/dL at 16 hours, the fluid was changed to dextrose-containing 0.45% sodium chloride while insulin was continued, allowing further correction of hypertonicity without an excessive fall in glucose.

Capillary glucose was checked hourly. Serum electrolytes, venous blood gas, and calculated effective osmolality were assessed every 2-4 hours during the initial phase (Table 2). Neurologic status, fluid balance, urine output, peripheral perfusion, and signs of thrombosis or rhabdomyolysis were assessed serially. Urine output remained above 1 mL/kg/hour after initial volume restoration.

**Table 2:** Serial biochemical response during the first 24 hours.

Time (h)	Glucose (mg/dL)	Na (mmol/L)	Effective osmolality (mOsm/kg)	K (mmol/L)	Phosphate (mmol/L)	Creatinine (µmol/L)
0	1,080	132	324	4.8	1.22	88
2	890	136	321.4	4.5	1.05	82
4	800	138	320.4	4.2	0.96	76
8	570	142	315.7	3.8	0.74	68
12	390	144	309.7	3.7	0.92	63
16	275	145	305.3	3.9	1.08	58
24	185	144	298.3	4.1	1.2	54
Reference	<200	135-145	275-295	3.5-5.1	0.9-2.0	40-90

Clinical outcome and transition to subcutaneous insulin

The child remained neurologically intact throughout treatment. Serum glucose fell at a controlled rate after insulin initiation, while measured sodium rose gradually as hyperglycemia corrected and effective osmolality declined from 324 to 298 mOsm/kg over 24 hours (Figure 1). Transient mild hypophosphatemia (nadir 0.74 mmol/L) was corrected through phosphate-containing potassium replacement. No seizures, cerebral injury, clinically significant hypokalemia, rhabdomyolysis, thrombosis, or acute kidney injury occurred.

**Figure 1:**
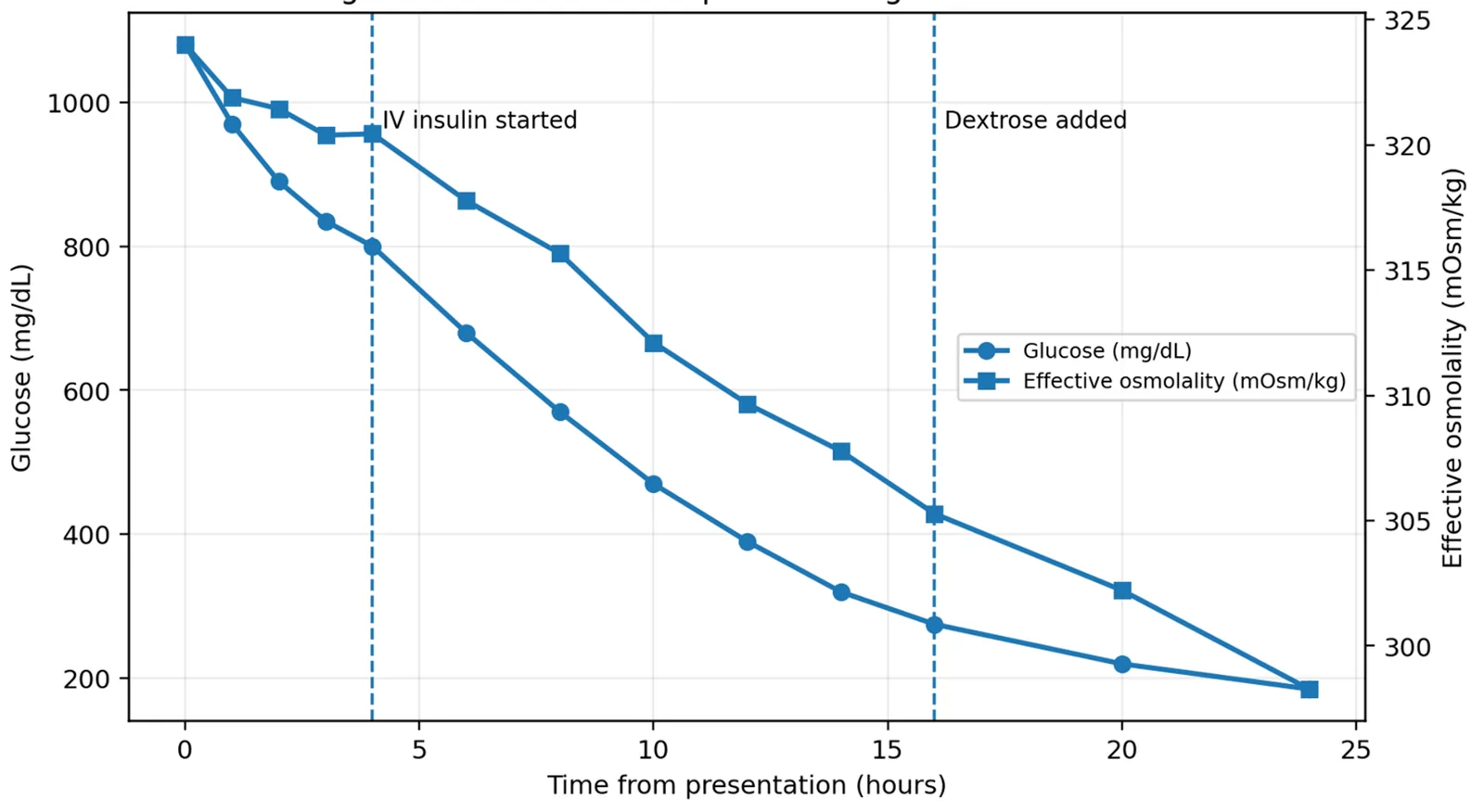
Glucose and calculated effective osmolality during the first 24 hours. Glucose and calculated effective osmolality during the first 24 hours. Insulin was started after four hours of fluid therapy, when the glucose decline with fluids alone slowed; dextrose was added when glucose reached 275 mg/dL.

At approximately 26 hours, the patient was clinically rehydrated, tolerating oral intake, and had stable electrolytes with an effective osmolality <300 mOsm/kg. A basal dose of insulin glargine was administered two hours before discontinuation of the intravenous insulin infusion, followed by a basal-bolus regimen using rapid-acting insulin with meals. The initial total daily insulin dose was approximately 0.6 units/kg/day and was subsequently individualized according to glucose monitoring, carbohydrate intake, and endocrinology review. Diabetes education, glucose monitoring education, hypoglycemia management, ketone testing, and sick-day rules were provided to the patient and family.

He was discharged home on hospital day 3 in good clinical condition. At the three-month follow-up, he had no recurrent hyperglycemic emergency, his weight had increased to 39.1 kg, and hemoglobin A1c had improved from 13.8% to 7.0%.

Etiologic and autoimmune evaluation

Blood samples for diabetes classification were obtained before the first dose of exogenous insulin for assays whose interpretation could be affected by insulin therapy. GAD65 antibody was strongly positive at 260.4 IU/mL. Insulin autoantibody was negative (<0.4 U/mL), and islet-cell antibody was negative (<1:10). C-peptide measured after initial stabilization was low at 0.8 ng/mL, with a concurrent glucose concentration of 286 mg/dL, supporting impaired endogenous insulin secretion in the setting of marked hyperglycemia. The combined biochemical phenotype and GAD65 positivity supported autoimmune T1DM despite negative results for the other measured autoantibodies (Table 3). The overall clinical course from symptom onset through three-month follow-up is summarized in Table 4.

**Table 3:** Diabetes classification and associated autoimmune screening. GAD65: Glutamic acid decarboxylase 65; TSH: Thyroid-stimulating hormone; T4: Thyroxine; IgA: Immunoglobulin A; tTG-IgA: Tissue transglutaminase immunoglobulin A.

Test	Result	Interpretation	Reference range
GAD65 antibody	260.4 IU/mL	Strongly positive	<5 IU/mL
Insulin autoantibody	<0.4 U/mL	Negative	<0.4 U/mL
Islet-cell antibody	<1:10	Negative	<1:4
C-peptide	0.8 ng/mL at glucose 286 mg/dL	Inappropriately low for hyperglycemia	0.8-3.9 ng/mL
TSH / free T4	2.1 mIU/L / 15.2 pmol/L	Normal	TSH: 0.5-4.3 mIU/L; free T4: 12.9-20.6 pmol/L
Total IgA	1.42 g/L	Normal	0.52-3.19 g/L
tTG-IgA	<2 U/mL	Negative	<4 U/mL

**Table 4:** Clinical timeline. HHS: Hyperosmolar hyperglycemic state; NaCl: Sodium chloride; HbA1c: Hemoglobin A1c.

Time	Clinical event
3 weeks before admission	Progressive polyuria, polydipsia, nocturia, fatigue, and weight loss.
0 h	ED presentation; glucose 1,080 mg/dL; effective osmolality 324 mOsm/kg; HHS diagnosed.
0-1 h	0.9% NaCl 20 mL/kg administered; perfusion improved.
4 h	Glucose 800 mg/dL; decline with fluids alone slowed to 35 mg/dL/h; insulin 0.05 units/kg/h started.
8 h	Glucose 570 mg/dL; effective osmolality 315.7 mOsm/kg; mild hypophosphatemia treated.
16 h	Glucose 275 mg/dL; effective osmolality 305.3 mOsm/kg; dextrose added to IV fluid.
24-26 h	Effective osmolality <300 mOsm/kg; clinically rehydrated; transitioned to subcutaneous insulin.
Day 3	Discharged after structured diabetes education.
3 months	HbA1c 7.0%; no recurrent hyperglycemic emergency.

Screening for associated autoimmune disease showed thyroid-stimulating hormone of 2.1 mIU/L, free thyroxine of 15.2 pmol/L, total IgA of 1.42 g/L, and tissue transglutaminase IgA <2 U/mL, with no biochemical evidence of autoimmune thyroid disease or celiac disease at diagnosis [6-11].

## Discussion

This case demonstrates a classic pediatric HHS biochemical phenotype occurring as the first presentation of autoimmune T1DM. The diagnostic distinction from DKA rests on the predominance of hyperosmolality and dehydration rather than ketoacidosis. The patient had extreme hyperglycemia, effective osmolality >320 mOsm/kg, preserved bicarbonate, venous pH above the pediatric HHS threshold, and minimal ketonemia. Mild acidemia alone should not be interpreted as DKA when bicarbonate and ketone measurements do not support significant ketoacidosis [2-12].

A key physiologic feature was the discrepancy between measured and corrected sodium. Severe hyperglycemia raises extracellular tonicity and shifts water from the intracellular to the extracellular compartment, lowering the measured sodium concentration. Although the measured sodium was 132 mmol/L, the corrected value, using the conventional 1.6 mmol/L correction factor, was approximately 148 mmol/L. During treatment, measured sodium appropriately increased as glucose fell, while effective osmolality declined progressively. This is a desirable pattern and illustrates why the direction of sodium change and serial effective osmolality are more clinically useful than a single uncorrected sodium value [2,12,13].

Fluid replacement is the cornerstone of pediatric HHS management. The estimated water deficit is often greater than in DKA, and volume restoration alone may cause a substantial initial decrease in glucose through improved renal perfusion and glucosuria. In this case, insulin was intentionally withheld during the initial fluid-responsive phase and started only after the hourly glucose decline slowed to below 50 mg/dL. This sequencing reduces the risk of an excessively rapid fall in glucose and osmolality. Once insulin was required, a lower infusion rate than that traditionally used for DKA was sufficient. Dextrose was subsequently introduced before complete normalization of glucose so that insulin could continue while the remaining hypertonicity was corrected gradually [2,4,13].

Electrolyte management is equally important because substantial total-body potassium and phosphate deficits may exist despite normal initial serum concentrations. Serial monitoring allowed early recognition of a mild phosphate nadir and maintenance of potassium within the desired range. Neurologic assessment, urine output, renal function, creatine kinase when clinically indicated, and surveillance for thrombosis are particularly relevant in pediatric HHS because of the degree of dehydration and hyperviscosity associated with the syndrome [2-5].

The autoimmune evaluation established the diabetes phenotype. Strong GAD65 antibody positivity and an inappropriately low C-peptide concentration in the presence of hyperglycemia supported autoimmune beta-cell failure. Negative insulin autoantibody or islet-cell antibody testing does not exclude T1DM because the sensitivity of individual autoantibodies varies, and no single marker is universally positive at diagnosis [6-8]. The lean phenotype in this patient reinforces that severe nonketotic hyperglycemia should not automatically be attributed to type 2 diabetes mellitus.

Previous pediatric reports have documented HHS as an initial manifestation of T1DM [3,4]. The present case reinforces that a lean phenotype does not exclude HHS or autoimmune T1DM and highlights the importance of integrating clinical dehydration, glucose kinetics, sodium physiology, effective osmolality, cautious insulin initiation, electrolyte replacement, and subsequent etiologic classification.

## Conclusions

HHS can be the initial presentation of autoimmune T1DM in a lean adolescent. Diagnosis requires recognition of severe hyperglycemia and effective hyperosmolality with minimal or absent ketoacidosis. Management should prioritize controlled restoration of intravascular volume, cautious introduction of low-dose insulin after initial fluid therapy, and serial assessment of glucose, sodium, effective osmolality, electrolytes, renal function, and neurologic status. Autoantibody testing and C-peptide measurement are valuable for clarifying the underlying diabetes phenotype. Early recognition of this uncommon presentation may help prevent complications associated with both severe dehydration and overly rapid correction of hypertonicity.
